# Genome‐wide identification of CpG island methylator phenotype related gene signature as a novel prognostic biomarker of gastric cancer

**DOI:** 10.7717/peerj.9624

**Published:** 2020-07-30

**Authors:** Zhuo Zeng, Daxing Xie, Jianping Gong

**Affiliations:** 1 Molecular Medicine Center, Tongji Hospital, Tongji Medical College, Huazhong University of Science and Technology, Wuhan, Hubei, China; 2 Department of GI Surgery, Tongji Hospital, Tongji Medical College, Huazhong University of Science and Technology, Wuhan, Hubei, China

**Keywords:** CpG island methylator phenotype, Prognostic signature, Gastric cancer, Overall survival

## Abstract

**Background:**

Gastric cancer (GC) is one of the most fatal cancers in the world. Results of previous studies on the association of the CpG island methylator phenotype (CIMP) with GC prognosis are conflicting and mainly based on selected CIMP markers. The current study attempted to comprehensively assess the association between CIMP status and GC survival and to develop a CIMP-related prognostic gene signature of GC.

**Methods:**

We used a hierarchical clustering method based on 2,082 GC-related methylation sites to stratify GC patients from the cancer genome atlas into three different CIMP subgroups according to the CIMP status. Gene set enrichment analysis, tumor-infiltrating immune cells, and DNA somatic mutations analysis were conducted to reveal the genomic characteristics in different CIMP-related patients. Cox regression analysis and the least absolute shrinkage and selection operator were performed to develop a CIMP-related prognostic signature. Analyses involving a time-dependent receiver operating characteristic (ROC) curve and calibration plot were adopted to assess the performance of the prognostic signature.

**Results:**

We found a positive relationship between CIMP and prognosis in GC. Gene set enrichment analysis indicated that cancer-progression-related pathways were enriched in the CIMP-L group. High abundances of CD8+ T cells and M1 macrophages were found in the CIMP-H group, meanwhile more plasma cells, regulatory T cells and CD4+ memory resting T cells were detected in the CIMP-L group. The CIMP-H group showed higher tumor mutation burden, more microsatellite instability-H, less lymph node metastasis, and more somatic mutations favoring survival. We then established a CIMP-related prognostic gene signature comprising six genes (*CST6, SLC7A2, RAB3B, IGFBP1, VSTM2L* and *EVX2*). The signature was capable of classifying patients into high‐and low‐risk groups with significant difference in overall survival (OS; *p* < 0.0001). To assess performance of the prognostic signature, the area under the ROC curve (AUC) for OS was calculated as 0.664 at 1 year, 0.704 at 3 years and 0.667 at 5 years. When compared with previously published gene-based signatures, our CIMP-related signature was comparable or better at predicting prognosis. A multivariate Cox regression analysis indicated the CIMP-related prognostic gene signature was an independent prognostic indicator of GC. In addition, Gene ontology analysis indicated that keratinocyte differentiation and epidermis development were enriched in the high-risk group.

**Conclusion:**

Collectively, we described a positive association between CIMP status and prognosis in GC and proposed a CIMP-related gene signature as a promising prognostic biomarker for GC.

## Introduction

Gastric cancer (GC) is responsible for over 1,000,000 new cases and around 783,000 deaths in the world annually, making it the 5th most frequently diagnosed cancer and the third leading cause of cancer-related death (Bray et al., 2018). Surgery with subsequent adjuvant chemoradiotherapy remains the only treatment with curative potential (Bang et al., 2012), and the prognosis for gastric adenocarcinoma is primarily determined by the TNM classification of staging system (Warneke et al., 2011). The clinical outcome, nonetheless, is notably variable and erratic in individual patient, which firmly implies that a few of the biological determinants of tumor behavior are unidentified. Thus, advances in molecular insight into GC are critically required for improved prognostic stratification and new targeted therapeutic strategies.

Recently, with the progress of high-throughput screening, sequencing has enabled a more thorough insight into the molecular identity of GC. An updated classification scheme has been introduced based on comprehensive molecular characterization including tumors infected with Epstein–Barr virus, tumors with microsatellite instability (MSI), and tumors with a distinct degree of aneuploidy, which were termed genomic stability and chromosomal instability. Each subgroup shows peculiar genetic and clinical characteristics (Cancer Genome Atlas Research, 2014).

Alterations of DNA methylation is a vital event during tumorigenesis, and gastrointestinal cancers show the highest frequency of DNA methylation alterations among the reported tumor types (Cancer Genome Atlas Research, 2014). Methylation of the dinucleotides of CpG islands throughout the genome is mediated by DNA methyltransferases (Craig & Bickmore, 1994), and commonly results in gene silencing. Disorder of DNA methylation in cancer affects gene expression and results in the cancer progression (Vaissiere, Sawan & Herceg, 2008).

CpG island methylator phenotype (CIMP) in tumors, which has been initially described and broadly debated in colorectal cancer (Hughes et al., 2013). Lately CIMP has been described in other tumor types including bladder, breast, glioblastoma, pancreatic and prostate cancers, as well as for gastric adenocarcinomas and is considered to be helpful for predicting prognosis (Jia et al., 2019; Moarii, Reyal & Vert, 2015; Ueki et al., 2000). In GC, conflicting conclusions regarding the prognostic association of CIMP have been scattered among previous studies, owing to the limitation of selected DNA methylation markers and the presence of multiple confounding factors in these studies (An et al., 2005; Ben Ayed-Guerfali et al., 2011; Park et al., 2010). Although a meta-analysis of the prognostic value of CIMP status in GC has been performed, an explicit conclusion was not reached (Powell et al., 2018).

In this study, we aimed to use publicly available data to comprehensively analyze CIMP in GC, and to develop a CIMP-related prognostic gene signature.

## Materials and Methods

### Data acquisition

We downloaded methylation data, which has 408,376 probes and 397 samples, including 395 GC samples and two normal samples, measured by the Illumina HumanMethylation450 platform, from the cancer genome atlas (TCGA)-STAD project (https://portal.gdc.cancer.gov/) by using the *TCGA-Assembler 2* package (Wei et al., 2018). RNA-Seq profiles were obtained from TCGA by virtue of *GDC Data Transfer Tool*. We downloaded two verified microarrays with matched clinical information from the GEO GC database: GSE13861 (Cho et al., 2011) (65 GC samples; platform: GPL6884 Illumina HumanWG-6 v3.0 expression beadchip ), GSE62254 (Cristescu et al., 2015) (300 GC samples; platform: GPL570 Affymetrix Human Genome U133 Plus 2.0 Array ). We used the *TCGAbiolinks* package to acquire the mutation data of GC samples (Colaprico et al., 2016). We obtained complete and matched clinical information on GC patients from cBioportal, including sex, age, histologic features, pathologic stage, family history and, infection status for *Helicobacter pylori* and Epstein–Barr virus.

Our study was performed according to the publication guidelines required by TCGA.

### Data analysis

The *Minfi* package was adopted to analyze methylation data (Aryee et al., 2011). In view of the distribution of CpG islands and the technical limitations of sequencing, we filtered the probes from the X and Y chromosomes or probes that are known to have common SNPs at the CpG site, and cross-reactive probes. The *DESeq2* package was adopted to analyze the differentially expressed genes (DEGs) between CIMP-related subgroups (Love, Huber & Anders, 2014). The criteria to determine DEGs were an adjusted *p*-value < 0.05 and an absolute value of log2 fold change >2, and BH method was used for adjustment for multiple testing. In order to identify the different pathways between GC samples with specific CIMP status, we performed GSEA analysis (Subramanian et al., 2005). The mRNA expression data downloaded from the Gene Expression Omnibus (GEO) database were normalized and analyzed by the *limma* package (Ritchie et al., 2015). The mutation data were summarized and analyzed by the *maftools* package (Mayakonda et al., 2018). Raw code for analyzing was uploaded in Data S1.

### Identification of CIMP in GC samples

To assess the CIMP feature in GC, CpG methylation sites with a relatively high variability of β-values in tumor samples (SD > 0.2) and relatively low β-values in normal samples (mean β value < 0.05), were chosen as the representative CpG methylation sites for subsequent clustering analysis, following a previous study (Li et al., 2019). The *ConsensusClusterPlus* package was adopted to run unsupervised clustering analysis based on M value of the selected 2,082 probes by means of the *K*-means algorithm (Wilkerson & Hayes, 2010).

### Analysis of tumor-infiltrating immune cells

The proportions of the 22 types of tumor-infiltrating immune cells were counted by CIBERSORT (Newman et al., 2015). CIBERSORT is a tool to provide an estimation of cell composition of mixed tissues based on gene expression profiles. We uploaded the modified gene expression data and standard annotation to the CIBERSORT portal and ran the LM22 signature, which contains 547 genes distinguishing 22 human immune cell types, and 1,000 permutations. Final results were normalized to sum up to one and could be assessed straightforwardly as cell fractions for contrast (Table S1).

### Development and validation of a CIMP-related prognostic signature

Using the *DESeq2* package, 1,072 DEGs were calculated between the CIMP-H and CIMP-L samples (Table S2), which were defined by relative methylation level. We then performed Cox regression to assess the prognostic significance of the DEGs. A suitable prognostic model is assumed to identify a smaller number of genes favorable for clinical practice. We therefore used the least absolute shrinkage and selection operator (LASSO) in combination to diminish the number of CIMP-related prognostic genes (Gui & Li, 2005). The *glmnet* package was used to perform the penalized Cox regression model with the LASSO penalty, and 1,000-times cross-validations were applied to determine the optimal values of the penalty parameter lambda. We selected lambda.min to get six CIMP-related prognostic genes. We then extracted the coefficients from multivariate Cox regression to build a gene signature. We adopted the *survminer* package to determine the cut-off value of the risk score. Then, the patients were divided into high-and low-risk subgroups according to their risk score. We used Kaplan–Meier analysis to compare OS rates between the high- and low-risk group. To identify whether the risk score was an independent factor, we conducted univariate Cox regression and multivariate Cox regression analyses. Statistical significance was inferred where *p* < 0.05.

### Validation in GEO dataset

To confirm the performance of our prognostic signature, we applied it to two GEO databases, GSE13861 (*n* = 65) and GSE62254 (*n* = 300). The mRNA expression data were prepared using the *limma* package. Scale, a generic R function including centering and scaling, was used to scale the GEO mRNA expression data to common range. Next we used a developed prognostic signature to calculate the risk score of every sample and divided patients by the cut-off value. Kaplan–Meier analysis was conducted between the high- and low-risk groups for overall survival. In addition, we conducted receiver operating characteristic (ROC) curve analysis and calculated an AUC for every database.

### Functional enrichment analysis

We applied the *clusterProfiler* package for Gene Ontology (GO) and Kyoto Encyclopedia of Genes and Genomes (KEGG) analysis between different risk score-related subgroups (Yu et al., 2012). Then we adopted the *GOplot* package to illustrate our GO and KEGG results (Tables S3 and S4). Protein–protein interaction analysis was carried out based on the DEGs of high-and low-risk subgroups using the STRING portal (https://string-db.org) and Cytoscape software (Shannon et al., 2003). Functional annotation of genes in the module was perform by DAVID database (Huang et al., 2007).

## Results

### Methylation landscape of GC sample

**Figure 1:**
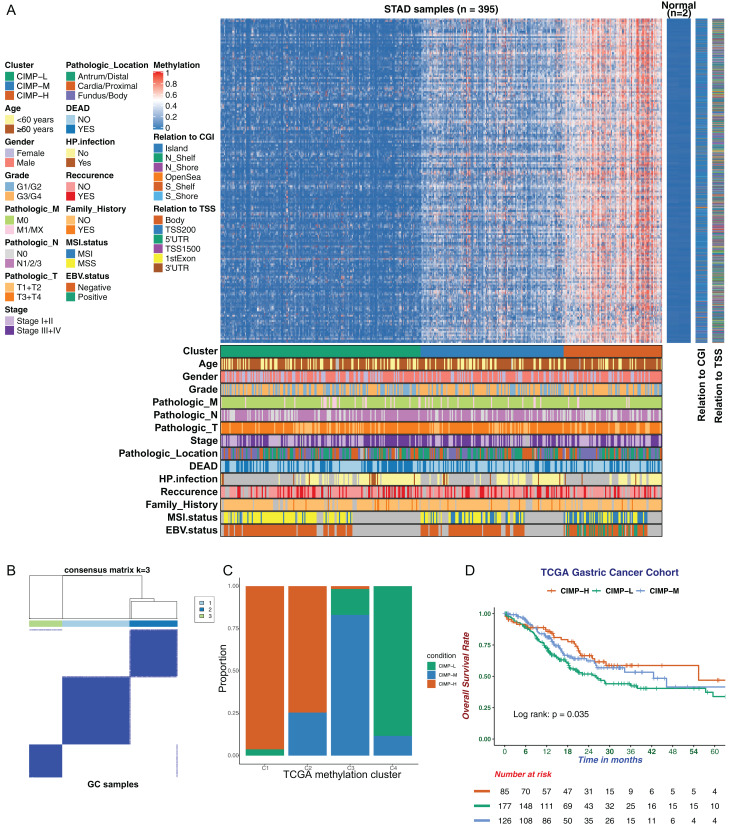
The landscape of CpG island methylator phenotype in gastric cancer. (A) Unsupervised hierarchical clustering of GC samples. The rows represent 2082 CpG methylation sites for clustering. Green, blue, and red cluster represents CIMP-Low (CIMP-L) subgroup, CIMP-Medium (CIMP-M) subgroup and CIMP-High (CIMP-H) subgroup respectively. The CIMP-L subgroup had the lowest methylation level. Clinical information is marked with different colors, and missing information is marked with gray. (B) Clustering result of K-means algorithm by *ConsensusClusterPlus*. (C) Comparison of CMIP-related subgroups with TCGA methylation cluster. (D) Kaplan-Meier survival curves of CIMP-related subgroups. The CIMP-H subgroup had a better OS than other subgroups.

In this study, we utilized DNA methylation profiles from TCGA database to perform a comprehensive analysis of DNA methylation in GC. We adopted 2,082 methylation sites with high variability as our CIMP signature for downstream analysis. Unsupervised hierarchical clustering analysis of 395 GC samples based on our specific CIMP signature was performed and all the patients were separated into three subgroups as CIMP-L, CIMP-M and CIMP-H (Figs. 1A and 1B; Table S5). The CIMP-L subgroup had the lowest methylation level, while the CIMP-H subgroup had broad hypermethylation across these sites. In addition, we plotted the Delta area and consensus CDF to verify our clustering pattern (Figs. S1A and S1B). To assess the performance of our classification based on CIMP signature, we reclassified GC samples according to a previous DNA methylation clustering analysis (Cancer Genome Atlas Research, 2014). A strong concordance was exhibited between these two classification systems (Fig. 1C). The C1 cluster, representing an EBV-associated DNA methylation signature with extreme hypermethylation, consisted primarily of CIMP-H samples, while the C4 cluster, representing a hypomethylated subgroup, consisted mainly of CIMP-L. Importantly, to evaluate the correlation between CIMP and prognosis, the overall survival of each subgroup was assessed by the Kaplan–Meier method. The result indicated a significant difference in prognosis among the different CIMP-related subgroups, with the CIMP-H group showing better prognosis and the CIMP-L group showing worse prognosis (Fig. 1D). In addition, we investigated the relationship between CIMP and progression-free survival (PFS). However, we found no significant differences in PFS existed among the CIMP-related subgroups (Fig. S1C).

The clinical characteristics of patients with different CIMP statuses were summarized (Table 1). Clinical features, including lymph node metastasis, MSI status and EBV infection, had significant differences between CIMP-related subgroups. No significant difference was found in aspects of age, gender, pathologic tumor classification, Lauren classification, grade or *Helicobacter pylori* infection. Specifically, within the CIMP-H subgroup more patients suffered MSI gastric adenocarcinoma and EBV infection, and less had lymph node metastasis. In addition, we found that in the CIMP-H subgroup, patients with EBV could be well distinguished from patients with MSI (Fig. S1D).

**Table 1:** Clinical and demographic characteristics of GC patients in CIMP-related subgroups.

	CIMP-L	CIMP-M	CIMP-H	*p*-Value
Number of patients	179	128	88	
Age (Mean (SD))	64.3 (10.5)	65.6 (10.5)	66.4 (11.3)	0.191
Gender				0.194
Female	70 (39.1%)	38 (29.7%)	28 (31.8%)	
Male	109 (60.9%)	90 (70.3%)	60 (68.2%)	
Pathologic_T				0.724
T1	7 (3.9%)	7 (5.5%)	7 (8.0%)	
T2	39 (21.8%)	24 (18.8%)	15 (17.0%)	
T3	85 (47.5%)	63 (49.2%)	38 (43.2%)	
T4	48 (26.8%)	34 (26.6%)	28 (31.8%)	
Pathologic_N				0.044
N0	51 (28.5%)	36 (28.1%)	37 (42.6%)	
N1	49 (27.4%)	31 (24.2%)	22 (25.3%)	
N2	36 (20.1%)	34 (26.6%)	9 (10.3%)	
N3	38 (21.2%)	27 (21.1%)	18 (20.7%)	
NX	5 (2.8%)	0 (0%)	1 (1.1%)	
Pathologic_M				0.115
M0	155 (86.6%)	115 (89.8%)	83 (94.3%)	
M1	11 (6.1%)	10 (7.8%)	2 (2.3%)	
MX	13 (7.3%)	3 (2.3%)	3 (3.4%)	
Pathologic_Stage				0.225
Stage I	23 (12.8%)	14 (10.9%)	16 (18.2%)	
Stage II	58 (32.4%)	42 (32.8%)	32 (36.4%)	
Stage III	78 (43.6%)	59 (46.1%)	38 (43.2%)	
Stage IV	20 (11.2%)	13 (10.2%)	2 (2.3%)	
Lauren.Class				0.112
Diffuse	49 (27.4%)	36 (28.1%)	18 (20.5%)	
Intestinal	115 (64.2%)	71 (55.5%)	61 (69.3%)	
Mixed	15 (8.4%)	21 (16.4%)	9 (10.2%)	
Grade				0.264
G1	4 (2.2%)	3 (2.3%)	2 (2.3%)	
G2	76 (42.5%)	41 (32.0%)	25 (28.4%)	
G3	94 (52.5%)	81 (63.3%)	60 (68.2%)	
GX	5 (2.8%)	3 (2.3%)	1 (1.1%)	
*H. pylori* infection				0.779
No	79 (87.8%)	56 (90.3%)	33 (91.7%)	
Yes	11 (12.2%)	6 (9.7%)	3 (8.3%)	
MSI.status				<0.001
MSI-H	4 (4.0%)	11 (14.4%)	34 (47.9%)	
MSI-L	11 (10.9%)	18 (23.7%)	8 (11.3%)	
MSS	86 (85.1%)	47 (61.9%)	29 (40.8%)	
EBV.positive				<0.001
Negative	101 (100%)	76 (100%)	46 (64.8%)	
Positive	0 (0%)	0 (0%)	25 (35.2%)	

### Gene set enrichment analysis in different CIMP-related subgroups

**Figure 2:**
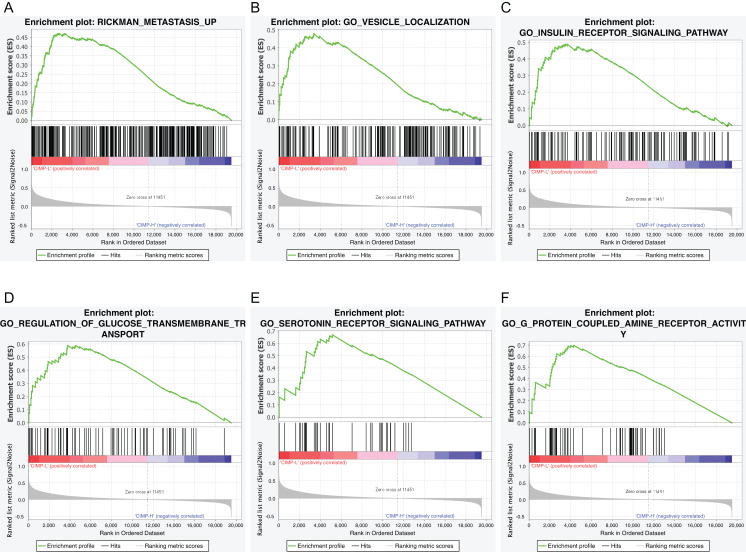
Gene set enrichment analysis of CIMP-related subgroups in the TCGA dataset. Significant enrichment in the CIMP-L subgroup compared with the CIMP-H subgroup. (A) RICKMAN metastasis up; (B) vesicle localization; (C) insulin receptor signaling pathway; (D) glucose transmembrane transport; (E) serotonin receptor signaling pathway; (F) G-protein coupled amine receptor activity.

To identify the biological processes or pathways potentially regulated by the CpG island methylation signature, we applied the GSEA analysis between different CIMP-related subgroups based on RNA-seq profiles. We found that the gene signatures of “Rickman metastasis up”, “Vesicle localization”, “Insulin receptor signaling pathway”, “Regulation of glucose transmembrane transport”, “Serotonin receptor signaling pathway”, “G-protein coupled amine receptor activity” were enriched in CIMP-L subgroup (Figs. 2A–2F). Importantly, all the pathways have been linked to GC progression.

### Tumor-infiltrating immune cells in different CIMP-related subgroups

**Figure 3:**
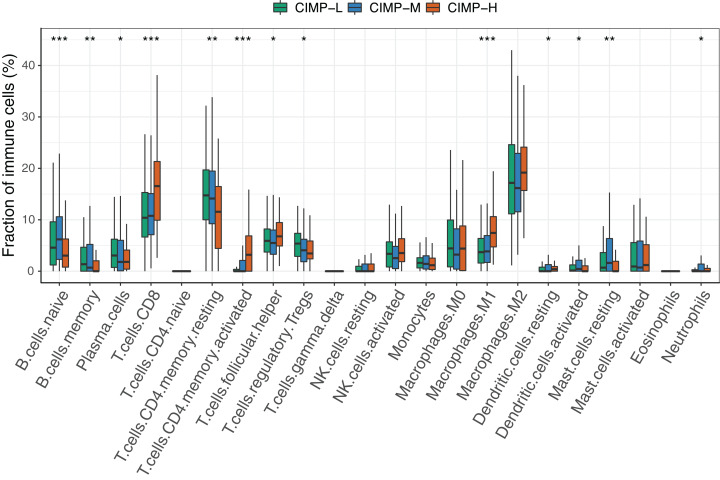
The comparison of fractions of tumor-infiltrating immune cells between CIMP-related subgroups in GC. (Kruskal–Wallis test was used, * represents for *p* < 0.05, ** represents for *p* < 0.01, *** represents for *p* < 0.001).

We assessed the presence of tumor-infiltrating immune cells (TIICs) in CIMP-related subgroups by using CIBERSORT (Fig. 3). Obviously, immune cells showed differential infiltration pattern between CIMP-related subgroups. The proportions of B cells, plasma cells, T cells CD4 memory resting, regulatory T cells and resting mast cells were significantly higher in the in the CIMP-L subgroup. Meanwhile, CD8+ T cells, T cells CD4 memory activated, T follicular helper cells, M1 macrophages, and Dendritic cells resting were higher in the CIMP-H subgroup. Other immune cells, including NK cells and monocytes, didn’t show significant differences. In addition, we found in most samples no apparent T cells CD4 naïve, T cells gamma delta and Eosinophils were infiltrated. These results indicated that CIMP-L subgroup have a distinct immune phenotype, which is considered to impair and suppress antitumor immunity.

### Analysis of DNA somatic mutations in patients with distinct CIMP status

**Figure 4:**
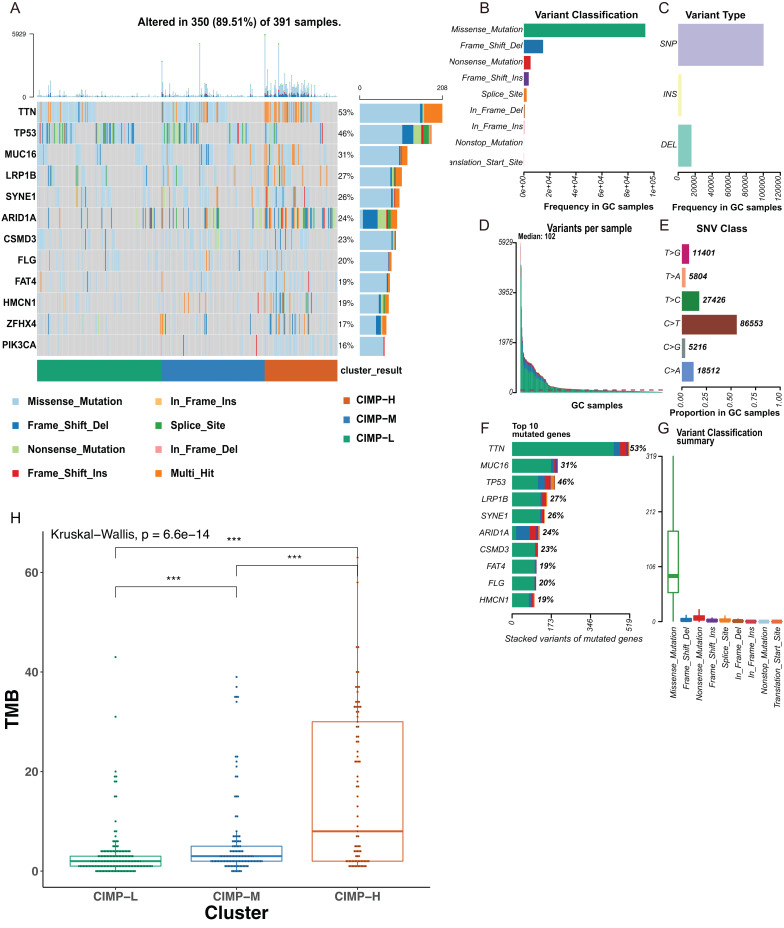
The mutational signatures in CIMP-related subgroups. (A) Waterfall plots showed mutation information of each gene in GC subgroups stratified by CIMP status, and various colors with annotations at the bottom represented the different mutation types. (B) Frequency of variant classifications. (C) Summary of variant types. (D) Summary of variants in per sample. (E) Summary of SNV classes. (F) Top ten mutated genes. (G) Summary of variant classifications. (H) Tumor mutation burden (TMB) of CIMP-related subgroups. The CIMP-H subgroup had higher TMB than the other subgroups. (Kruskal–Wallis test was used, SteelDwass test was used for post-hoc test, * represents for *p* < 0.05, ** represents for *p* < 0.01, *** represents for *p* < 0.001).

A distinct set of genetic aberrations between CIMP-related subgroups was evident in our study. We found 350 samples with mutations in a total of 391samples (89.51%), with TTN and TP53 ranking as the most common mutation gene (Fig. 4A). Mutation of the TP53 gene was found enriched in the CIMP-L subgroup. At the same time, mutations of TTN and MUC16 were higher in the CIMP-H subgroup. The most common mutations in GC samples were missense mutations, comprising the majority of SNPs, the main SNV classification was C > T transition, and the number of altered bases in each sample was counted (Figs. 4B–4E). We then showed the top 10 mutated genes in GC with ranked percentages, including TTN (53%), MUC16 (31%), TP53 (46%), LRP1B (27%), SYNE1 (26%), ARID1A (24%), CSMD3 (23%), FAT4 (19%), FLG (20%), HMCN1 (19%), and summarized the mutation types in GC (Figs. 4F and 4G). We then calculated the tumor mutation burden (TMB), which is considered to correlate with enhanced clinical response to immunotherapy and superior OS. We found TMB was higher in the CIMP-H subgroup (Fig. 4H).

### Establishment of a CIMP-related prognostic gene signature

**Figure 5:**
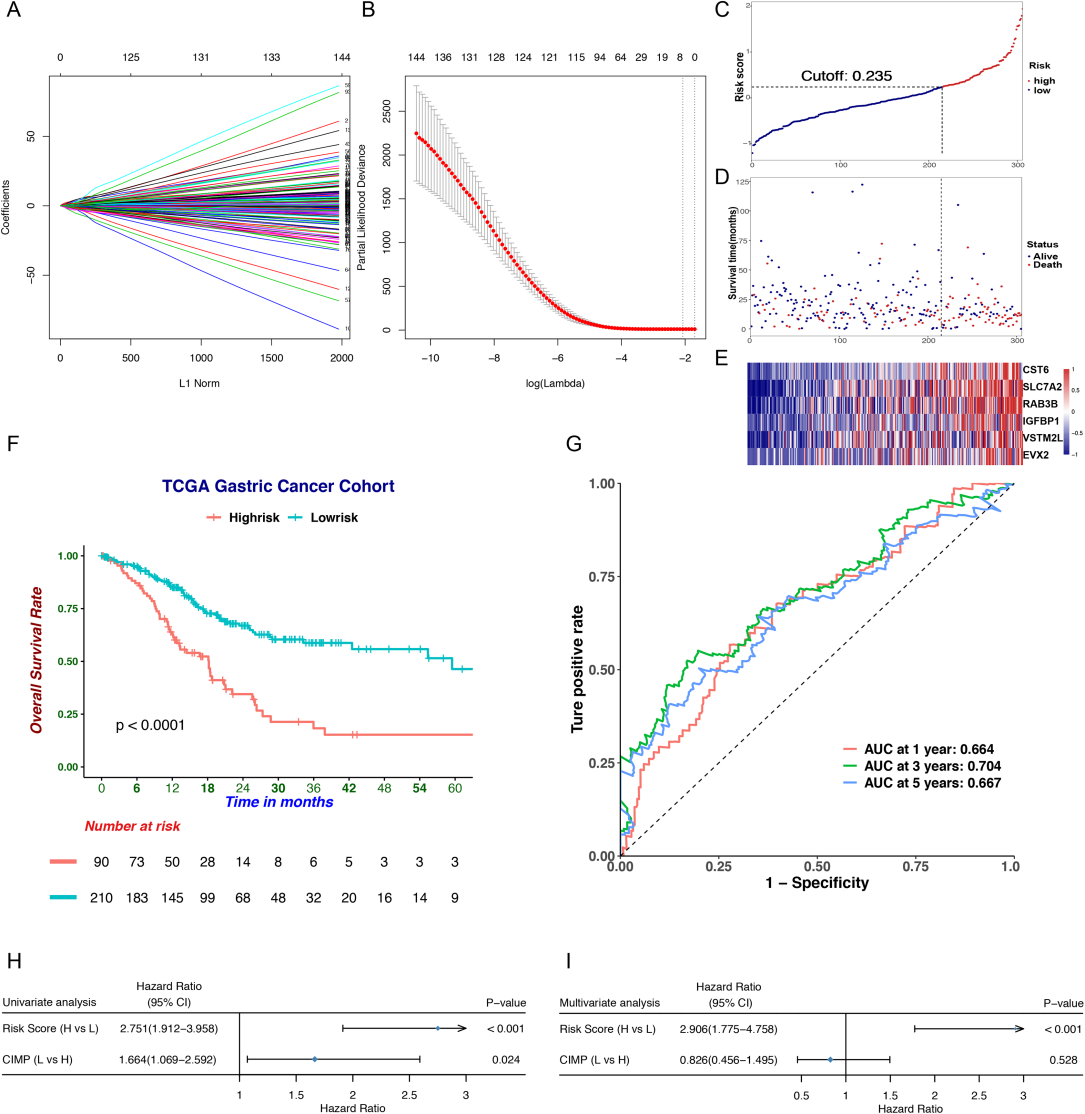
Prognostic analysis of the CIMP-related prognostic gene signature in TCGA cohort. (A) LASSO coefficient profiles of candidate genes. Each curve indicated one gene. (B) Cross-validation in the LASSO model. (C) The distribution of risk score. (D) The distribution of survival status. (E) The distribution of expression levels of the six genes in TCGA cohort. (F) The Kaplan–Meier curve for patients divided into high-and low-risk based on CIMP-related prognostic gene signature. (G) Receiver operating characteristic curve of CIMP-related prognostic signature at different years. (H) and (I) Univariate and multivariate regression analysis of the CIMP status and risk score calculated based on CIMP-related prognostic signature.

To screen differentially expressed genes (DEGs) in CIMP subgroups, we downloaded RNA-seq data for 208 samples defined as CIMP-H or CIMP-L subgroups from the TCGA database and analyzed the data by using the *DEseq2* package. We identified 1,072 DEGs, which we narrowed down to 147 genes highly associated with OS using univariate Cox regression. To obtain the genes with the highest potential prognostic values, we used least absolute selection and shrinkage operator (LASSO) regression analysis. A prognostic signature comprising six genes, including *cystatin E/M (CST6), solute carrier family 7 member 2 (SLC7A2), RAB3B, member RAS oncogene family (RAB3B), insulin like growth factor binding protein 1 (IGFBP1), V-set and transmembrane domain containing 2 like (VSTM2L)* and *even-skipped homeobox 2 (EVX2)*, was developed (Figs. 5, 1A and 1B; Table 2). The risk score was calculated as follows: risk score = (0.230 × the normalized expression of *CST6*) + (0.257 × the normalized expression of *SLC7A2*) + (0.156 × the normalized expression of *RAB3B*) + (0.114 × the normalized expression of *IGFBP1*) + (0.024 × the normalized expression of *VSTM2L*) + (0.187 × the normalized expression of *EVX2*). The cutoff value (0.235) was counted by the *survminer* package (Fig. 5C). The patients were then divided into high- and low-risk subgroups according to their risk score. We found high-risk patients had more deaths and higher expression levels of CIMP-related prognostic genes (Figs. 5D and 5E). We then found those in the high-risk had a worse OS than those in the low-risk group (Fig. 5F). To access the performance of the prognostic signature, time-dependent ROC curves and AUC were printed and counted (Fig. 5G). The AUC was 0.664 at 1 year, 0.704 at 3 years and 0.667 at 5 years. The univariate and multivariate Cox regression analyses indicated that the predictive value of risk score for overall survival was independent of CIMP status (Figs. 5H and 5I).

**Table 2:** The CIMP-related prognostic gene signature based on six genes in TCGA cohort. HR: hazard ratio, CI: confidential interval.

		Univariate Cox regression			
Symbol	Multivariate Cox regression coefficient	HR	95%CI	*p*-Value	z score
CST6	0.230	1.327	[1.139–1.547]	2.80E−04	3.633
SLC7A2	0.257	1.405	[1.192–1.656]	5.01E−05	4.055
RAB3B	0.156	1.361	[1.152–1.607]	2.76E−04	3.637
IGFBP1	0.114	1.244	[1.070–1.448]	4.63E−03	2.832
VSTM2L	0.024	1.233	[1.053–1.444]	9.44E−03	2.596
EVX2	0.187	1.221	[1.084–1.376]	1.02E−03	3.284

### Validation and evaluation of the CIMP-related signature in the GEO cohort

**Figure 6:**
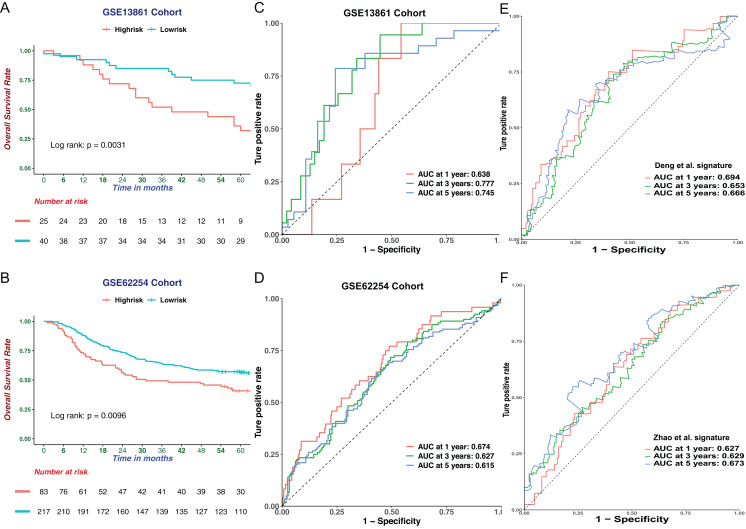
Validation of the CIMP-related prognostic gene signature in independent cohorts. The Kaplan–Meier curve for patients divided into high-and low-risk based on CIMP-related prognostic gene signature in (A) GSE13861 (*n* = 65) and (B) GSE62254 (*n* = 300) cohorts. Receiver operating characteristic curve of CIMP-related prognostic signature at different years in (C) GSE13861 (*n* = 65) and (D) GSE62254 (*n* = 300) cohorts. (E) and (F) Receiver operating characteristic curves of the other signatures reported in previous studies in the prediction of OS for TCGA cohort.

To further verify the robustness of the six-genes prognostic signature in GC, two verified microarrays with matched clinical information from the GEO GC database were analyzed. In every dataset, patients were stratified into high-or low-risk group according to the cutoff point calculated following the prognostic signature. Consistent with the results from the TCGA cohort, the high-risk group had a worse survival outcome in two datasets (Figs. 6A and 6B). Indicating favorable performance of our prognostic signature, the AUC of the GSE13861 dataset was 0.638 at 1 year, 0.777 at 3 years, 0.745 at 5 years (Figs. 6C). The AUC of GSE62254 dataset was 0.674 at 1 year, 0.627 at 3 years and 0.615 at 5 years (Fig. 6D). We then compared our CIMP-related prognostic signature with two prognostic signatures published previously. We extracted formulae from each study, and the results of ROC curve analysis implied that the CIMP-related prognostic signature was comparable or better at predicting the prognosis in the TCGA cohort (Figs. 6E and 6F). Taken together, the prognostic signature based on CIMP was a reliable prognostic marker in GC.

### The risk score developed from the six genes signature as an independent prognostic factor

**Figure 7:**
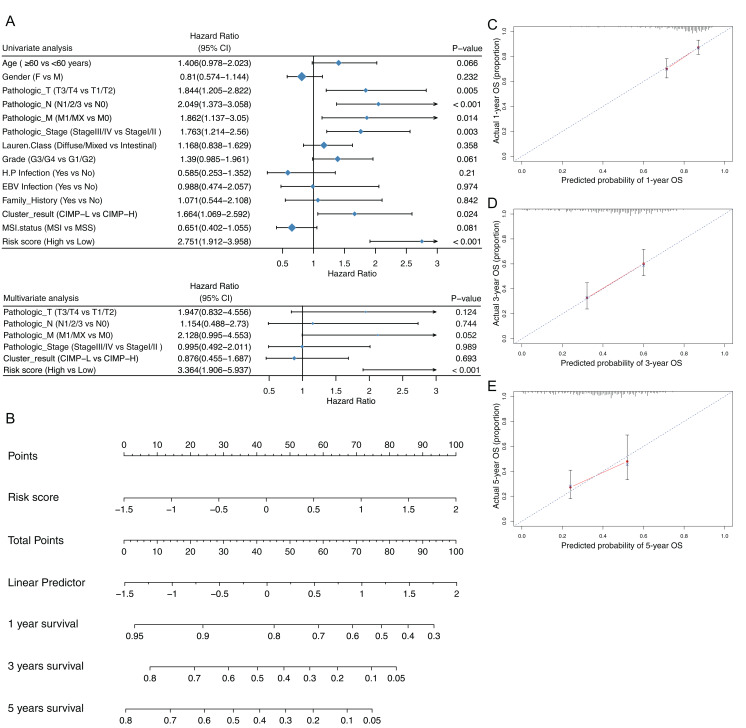
Prediction of risk score for overall survival (OS). (A) Univariate and multivariate regression analysis of the relation between the CIMP-related prognostic risk score and clinicopathological characters regarding OS (CI, confidential interval). (B) The nomogram for predicting probabilities of overall survival at 1,3 and 5 years. Calibration plot of predicted survival and actual survival at (C) 1 year, (D) 3 years, (E) 5 years.

**Figure 8:**
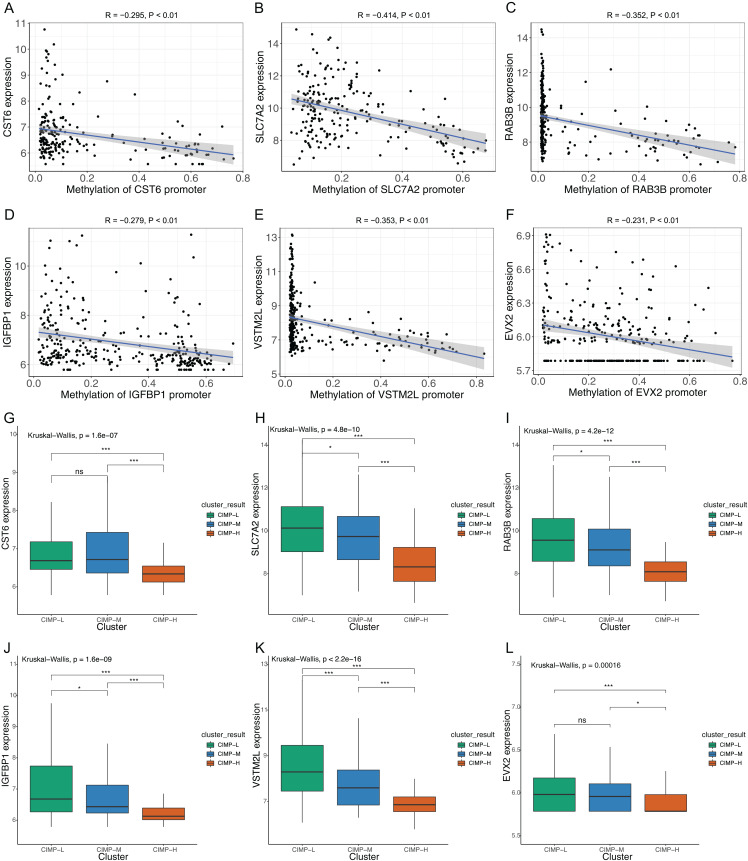
Correlations of CIMP-related prognostic gene signature. Correlations between signature gene promoter methylation and signature gene expression (A) CST6; (B) SLC7A2; (C) RAB3B; (D) IGFBP1; (E) VSTM2L; (F) EVX2 (Pearson’s rank correlation analysis was used). Expression of prognostic signature genes in CIMP-related subgroups (G) CST6; (H) SLC7A2; (I) RAB3B; (J) IGFBP1; (K) VSTM2L; (L) EVX2 (Kruskal-Wallis test was used, SteelDwass test was used for post-hoc test, * represents for *p* < 0.05, ** represents for *p* < 0.01, *** represents for *p* < 0.001, ns represents no significance).

We contrasted the prognostic value of the risk score was contrasted with clinical parameters by univariate and multivariate analyses. Clinical parameters included diagnostic age, gender, pathologic TNM, pathologic stage, pathologic grade, Lauren classification, status of *H. pylori* and EB virus infection and MSI status. We found that risk score acted as an independent prognostic factor and had significant effects in both the univariate analysis and the multivariate analysis, with *p* values < 0.05 (Fig. 7A). Furthermore, the risk score had robust prognostic value (with HR = 3.364, 95% CI [1.906–5.937]). We then used the risk score as a nomogram to predict patients’ outcome (Fig. 7B). The Calibration plot indicated that predicted OS and the actual OS rates at 1,3 and 5 years were similar (Figs. 7C–7E). To verify the role of methylation in the expression of prognostic signature gene, we used Pearson correlation to evaluate the relationship between the methylation levels of the *CST6, SLC7A2, RAB3B, IGFBP1, VSTM2L* and *EVX2* promoters and their expression levels. Consistent results were found among these six genes (Figs. 8A–8F). Moreover, the expression of signature genes was consistent among the CIMP-related subgroups. Expression levels of the signature genes were higher in the CIMP-L subgroup than those in the other subgroups (Figs. 8G–8L).

### Distinct biological processes in risk score stratified subgroups

**Figure 9:**
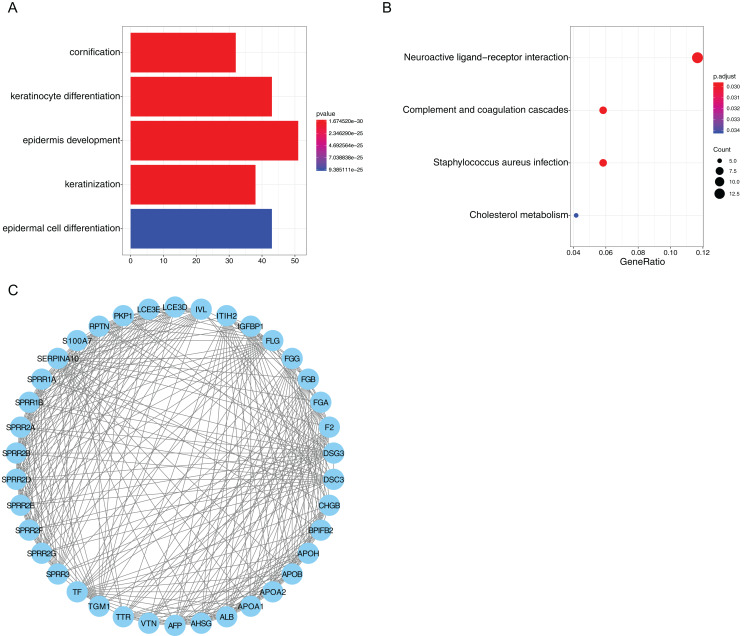
Functional enrichment analysis of risk score related genes. (A) GO analysis of differentially expressed genes between risk score stratified subgroups. (B) KEGG analysis of differentially expressed genes between risk score stratified subgroups. (C) The most significant module identified in the PPI network of differentially expressed genes between risk score stratified subgroups.

We identified 382 DEGs between the high-risk and low-risk subgroups in GC samples. Then, we carried out GO and KEGG analyses to identify the molecular mechanisms associated with these DEGs. For GO analysis, the top five enriched terms were “cornification”, “keratinocyte differentiation”, “epidermis development”, “keratinization” and “epidermal cell differentiation” (Fig. 9A). In KEGG analysis four pathways were enriched, including “neuroactive ligand-receptor interaction”, “complement and coagulation cascades”, “staphylococcus aureus infection” and “cholesterol metabolism”. The “neuroactive ligand-receptor interaction” was shown to be the main associated pathway with 14 genes involved (Fig. 9B). In addition, STRING was used to draw 382 DEGs into a PPI network complex, which contained 366 nodes and 1,176 interactions (Fig. S3). Then, Cytoscape was used to identify the most significant module in the PPI network. The most significant module (score = 17.2) recognized by MCODE, a plug-in of Cytoscape, contained 38 nodes and 318 interactions (Fig. 9C). Consistent with the results of GO analysis, the genes in the module were found to be related to “keratinocyte differentiation”, “keratinization”, “peptide cross-linking” and “epidermis development” (Table 3).

**Table 3:** The enriched GO terms of genes in the most significant module.

ID	Term	Count	*p*-Value
GO:0030216	keratinocyte differentiation	15	5.49E−25
GO:0031424	keratinization	13	3.49E−23
GO:0018149	peptide cross-linking	13	6.06E−23
GO:0008544	epidermis development	11	7.52E−16
GO:0002576	platelet degranulation	8	1.25E−09

## Discussion

In the field of cancer research, increasing attention has focused on DNA methylation. Patterns of DNA methylation can predict prognosis and survival of human cancers (Hao et al., 2017). CIMP refers to promoter CpG island hypermethylation and is well characterized in colorectal cancers. In contrast, the relationships between CIMP and clinicopathological features are controversial in GC. Previous studies on CIMP used different methods to measure methylation, like measuring across several CpG sites of a gene or across several genes. Conflicting conclusions may be drawn, due to variation among studies in methodologies of DNA methylation analysis and CIMP marker panels, which brought the bias of chosen marker panels (An et al., 2005; Oue et al., 2003; Park et al., 2010; Powell et al., 2018). In the present study, we used the methylation data measured by the Illumina HumanMethylation450K platform to assess the DNA methylation status of 40,8376 CpG sites including CpG sites located at the promoter regions of protein-coding genes in multiple samples simultaneously. We adopted methylation sites with high variability as our CIMP signature for unsupervised hierarchical clustering to comprehensively assess CIMP status in GC. In addition, our study included more patients with methylation data from the same platform than previous study (Cancer Genome Atlas Research, 2014). We firstly used this methodology to divide GC samples into three distinct subgroups according to their levels of methylation at selected methylation sites and describe the positive relation between CIMP and prognosis at the global methylation level, in contrast to analyses using chosen markers. Consistent with this, our CIMP-H subgroup showed more favorable clinical characteristics including less lymph node metastasis and lower metastasis status. Promoter hypermethylation is a prominent feature of EBV-associated GC, and we found that samples with EBV infection were enriched in the CIMP-H subgroup in this study (Kang et al., 2002). Previous studies indicated that GC patients with MSI show a significant longer overall survival compared with those who have MSS and assumed that MSI GC has a better prognosis because of its earlier stage at diagnosis, less lymph node metastasis and intestinal histological type (Mathiak et al., 2017; Polom et al., 2018). Consistent with these researches, samples with MSI were enriched in the CIMP-H subgroup which showed a better prognosis in our study. We then plotted the Kaplan–Meier survival curves according to previous DNA methylation clustering. However, we found no significant differences in this clustering (Fig. S2A). We believed that it was caused by too few cases in the C1 group, which was corresponding to our CIMP-H group. Therefore, we combined C2 and C3 groups, and plotted Kaplan-Meier survival curves between C2 + C3 and C4 groups. We found that the difference in survival was still not statistically significant, but we could see the difference in survival between these two groups (Fig. S2B). We believed it was due to the fact that the previous DNA methylation clustering was based on the merger of two methylation platforms, and that the number of samples included was not as large as ours. In our study, we found that there was no significant relationship between CIMP and PFS in GC. However, considering that some patients lack PFS information, we need to include more patients with complete PFS information to clarify the role of CIMP in PFS of GC in future research.

Our study provided insight into the landscapes of molecular features in patients with distinct CIMP statuses. Based on the gene set enrichment analysis, we identified cancer-related oncogenic pathways enriched in the CIMP-L subgroup, including metastasis, vesicle transfer, G-protein coupled receptor, energy transfer. Cancer cell-derived vesicles serve as intercellular communication vehicles and carry pathogenic components, such as proteins, mRNA, miRNA, DNA, lipids and transcriptional factors, that can mediate paracrine signaling in the tumor microenvironment (Fujita, Yoshioka & Ochiya, 2016). Vesicles mediate the formation of pre-metastatic niches to promote metastasis in tumors, including GC (Deng et al., 2017; Jung et al., 2009; Peinado et al., 2012). Li et al. (2011) reported that Insulin-like growth factor-I (IGF1) regulated the expression of the VEGF ligand to facilitate angiogenesis and lymphangiogenesis in GC cell lines, and blocking IGF1 could enhance the effectiveness of bevacizumab. High glucose conditions were shown to promote GC cell proliferation and reduce susceptibility to chemotherapy (Zhao et al., 2015). In addition, serotonin-induced signaling pathways promoted tumor progression (Sarrouilhe & Mesnil, 2019). This suggests these pathways could have potential as novel drug targets.

Immunotherapy is becoming a routine cancer treatment option, and disparate tumor-infiltrating immune cells profiles were observed among CIMP-related subgroups. CD8+ (cytotoxic) T cells are very important for immune defense and tumor surveillance, and are known to correlate with more favorable outcome in GC (He et al., 2017). In GC, tumor-associated plasma cells are polarized to produce IgG4 and associated with tumor progression and poor prognosis (Miyatani et al., 2016). Regulatory T cells (Tregs) are T cells which have a role in regulating or suppressing other cells in the immune system, leading to limiting excessive immune responses. Tregs suppress activation, proliferation and cytokine production of CD4+ T cells and CD8+ T cells. Tregs are thought to suppress B cells and dendritic cells. Liu et al. (2019) revealed that Tregs promoted Lgr5 expression in GC cells via TGF-β1 signaling pathway and was negatively associated with survival. Differences in the levels of the M1 macrophages were also observed. M1 macrophages are an integral cellular component of the immune system, and play a critical role in protection against intracellular pathogens and cancer cells (Yin et al., 2017). M1 macrophages have been reported to inhibit tumor growth in GC (Liu et al., 2013).

Recently, TMB has been increasingly accepted as a biomarker of response to immunotherapy. High TMB contributes to the synthesis of aberrant and potentially immunogenic mutation-associated neoantigens by the cancer cells, which attract CD8+ CTLs and activated Th1 cells to the tumor microenvironment. In this study, we found higher TMB in the CMIP-H subgroup, potentially indicating a better response to immunotherapy in this group. Consistent with the distribution of TMB, more MSI samples, especially MSI-H, were detected in the CIMP-H subgroup. The prevalence of MSI in GC is relatively high, and as MSI-H GCs are strongly associated with PD-L1 positivity, they could be applicable targets of anti-PD-1 therapies (Kim et al., 2018). The mutations of TTN and MUC16 were announced to be correlated with better survival result in lung and GCs (Cheng et al., 2019; Li et al., 2018a).

Recent researches have indicated that clinicopathological parameters such as tumor depth, lymph node metastasis, margin status, and metastatic condition are unsatisfactory for accurately predicting patient prognosis. Outcome varies tremendously among patients with comparable clinicopathological features. Therefore, with the advantage of high-throughput sequencing technologies, mRNAs have been used as molecular biomarkers of the cancer diagnosis and prognosis and shown their critical clinical application potential. For examples, Zhao et al. (2019) investigated genes relevant to the cell cycle from the TCGA database and described a set of five genes (*MARCKS, CCNF, MAPK14, INCENP* and *CHAF1A*), which were significantly associated with OS. They used this signature to stratify GC patients into two groups with significantly different survival outcomes. Distinct clinical features were also demonstrated between the two groups. In another research, the predictive value of DNA methylation gene for prognosis was determined in GC, and different pathways and biological processes associated with tumorigenesis were found in groups with distinct gene methylation levels (Hu et al., 2019).

In our study, a gene signature including *CST6, SLC7A2, RAB3B, IGFBP1*, *VSTM2L* and *EVX2*, was developed based on CIMP. *CST6* has been reported to play a role in the progression of triple-negative breast cancer (TNBC) and may act as a tumor-promoter gene. High *CST6* expression was also associated with a higher rate of lymph node metastasis (Li et al., 2018b). *SLC7A2* is essential for transport of L-arginine, lysine and ornithine and genetic polymorphisms in the *SLC7A2* gene are associated with colorectal cancer progression (Sun et al., 2017). *SLC7A2* has also been found to play a role in radio-resistance of non-small cell lung cancer. *RAB3B*, a member of RAS oncogene family, is shown to be a target of miR-200b, which is supposed to be tumor suppressor in GC (Tang et al., 2013; Ye et al., 2014). *RAB3B*, has been shown to be overexpressed in prostate cancer patients and promote prostate cancer cell survival (Tan et al., 2012). *IGFBP1*, an insulin-like growth factor binding protein, is revealed to be associated with hematogenous metastasis and poor survival in GC. And the expression of *IGFBP1* is positively associated with tumor invasion, lymph node metastasis and vascular invasion (Sato et al., 2019). *VSTM2L* is reported to be downregulated in the *H. pylori*-positive GC samples (Hu et al., 2018). However, the role of *VSTM2L* in tumors is rarely reported, and its role requires further studies. *EVX2* is recently revealed to be regulated by methylation and serve as a methylation biomarker for lung cancer (Rauch et al., 2012). In our study, the expression of *EVX2* was higher in CIMP-L subgroup consistent with its mechanism of epigenetic regulation. Our six-genes risk signature was an independent prognostic biomarker of GC, with patients in high-risk groups showing significantly worse prognosis than those in low-risk groups. Our results support the notion that gene risk signature might have more predictive power than traditional prognostic parameters. The prognostic performance of our signature was validated in the TCGA dataset, and the external datasets GSE13861 and GSE62254. Further, it compared favorably to two other gene-based signatures (Deng et al., 2018; Zhao et al., 2019), based on ROC and AUC analyses predicting 1-, 3- and 5-year overall survival. In our study, distinct biological analyses in risk score stratified subgroups indicated that keratinization and keratinization-related processes may play an important role in GC progression. At present, there is no research about the role of keratinization in GC and it needs further studies. In addition, the neuroactive ligand-receptor interaction was shown to be the most significant pathway in stratified subgroups and revealed to be involved in apoptosis and cell proliferation (Zan & Li, 2019).

## Conclusion

In summary, we first identified an accurate and comprehensive association between CIMP and clinical prognosis in GC, where high CIMP indicated better patient prognosis. We then developed and validated a six-genes prognostic signature related to CIMP that can predict the survival of patients with GC, where higher risk score indicated worse patient prognosis. This signature could be an effective tool in clinical practice as a supplement to traditional staging system to indicate progression and predict overall survival of GC. However, our study has some limitations. Firstly, it was based on a retrospective design, so the numbers of patients with the same clinical features in CIMP subgroups were not comparable. Also, the number of datasets used to validate the clinical prognostic signature is not large, and further validation by future prospective studies is desirable. Knowledge of the signature genes in GC development is currently scarce and further experiments are needed to verify their potential molecular mechanisms.

## Supplemental Information

10.7717/peerj.9624/supp-1 Supplemental Information 1 Unsupervised hierarchical clustering analysis of GC samples. (A) Delta area of the consensus clustering. (B) Consensus cumulative distribution function (CDF) of the consensus clustering. (C) Progression-free survival curves of CIMP-H and CIMP-L subgroups. (D) MSI status in patients with EBV in CIMP-H subgroup.

10.7717/peerj.9624/supp-2 Supplemental Information 2 The relationship between prognosis and DNA methylation clustering reported by Cancer Genome Atlas Research. (A) Kaplan-Meier survival curves of the C1, C2, C3 and C4 groups. The C1 group has the most prevalence of DNA hypermethylation and the C4 group has the lowest methylation level. The C2 and the C3 group have the medium methylation level. (B) Kaplan-Meier survival curves of the C2 + C3 and C4 groups. The C2 + C3 group represents the combination of the C2 and C3 groups.

10.7717/peerj.9624/supp-3 Supplemental Information 3 The PPI network of differentially expressed genes between risk score stratified subgroups. The PPI network was analyzed by String software. 366 nodes and 1176 edges are in the PPI network.

10.7717/peerj.9624/supp-4 Supplemental Information 4 Tumor-infiltrating immune cells profile.

10.7717/peerj.9624/supp-5 Supplemental Information 5 Differentially expressed genes between CIMP-H and CIMP-L groups.

10.7717/peerj.9624/supp-6 Supplemental Information 6 The result of GO analysis.

10.7717/peerj.9624/supp-7 Supplemental Information 7 The result of KEGG analysis.

10.7717/peerj.9624/supp-8 Supplemental Information 8 The result of cluster in TCGA cohort.

10.7717/peerj.9624/supp-9 Supplemental Information 9 Raw code.

## Additional Information and Declarations

### Data Availability

The following information was supplied regarding data availability: The raw data are available from TCGA project TCGA-STAD and from the NCBI GEO GC database (accession numbers: GSE13861, GSE62254). The code used to analyze the data is available as a Supplemental File.
