# Supplementary figures and images for: Genome‐wide identification of CpG island methylator phenotype related gene signature as a novel prognostic biomarker of gastric cancer
Source: PeerJ. 2020 Jul 30;8:e9624. doi: 10.7717/peerj.9624 (PMC7396145; doi:10.7717/peerj.9624)

A

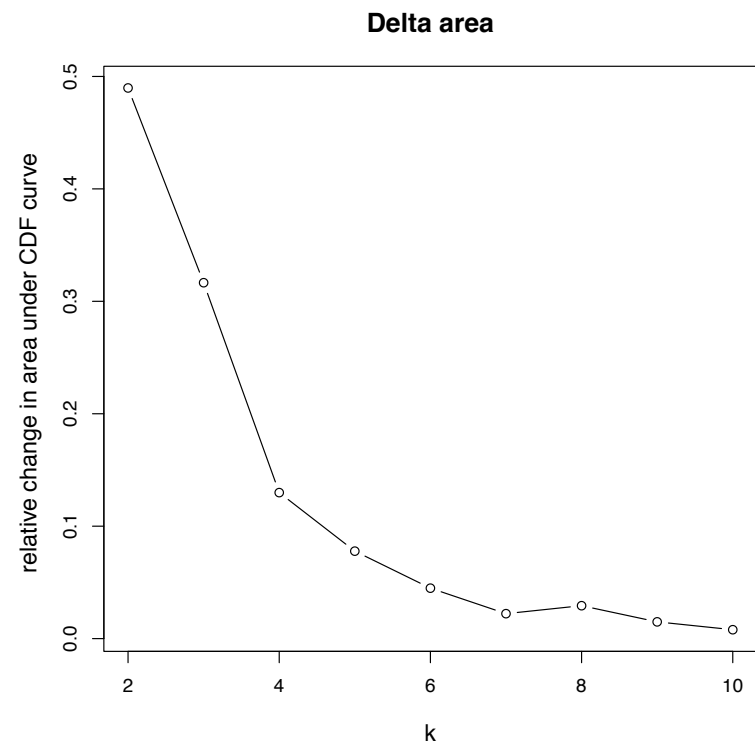

B

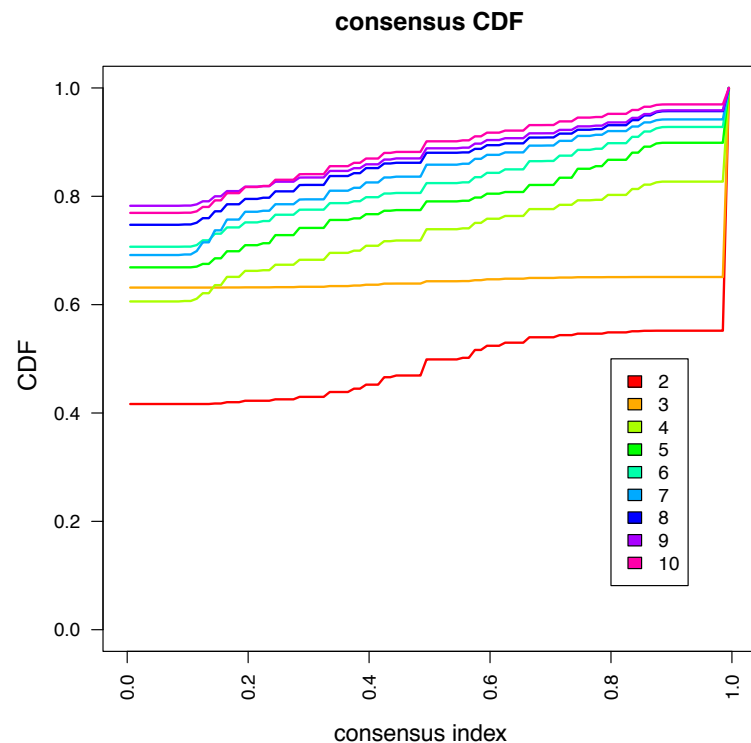

C

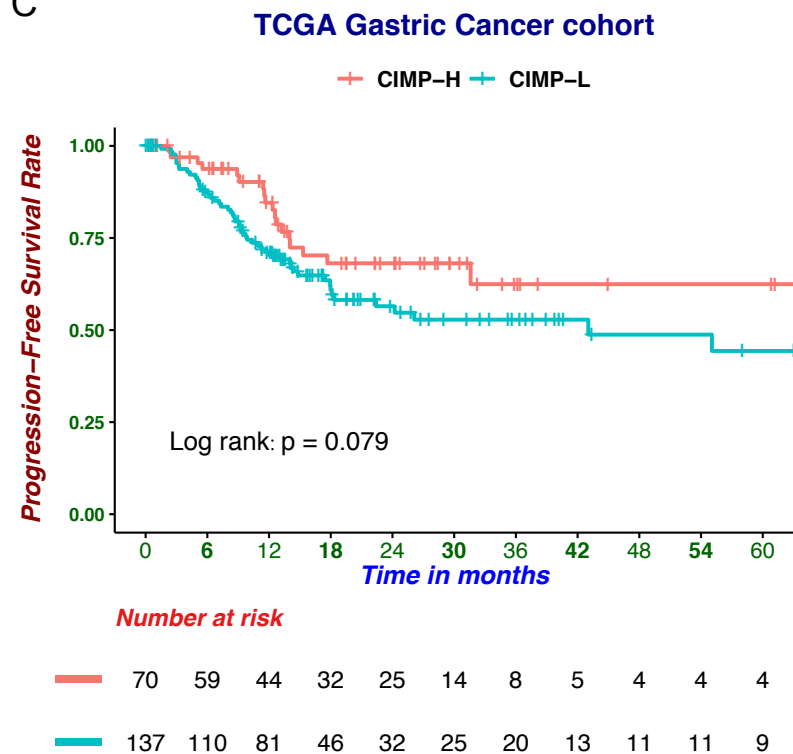

D

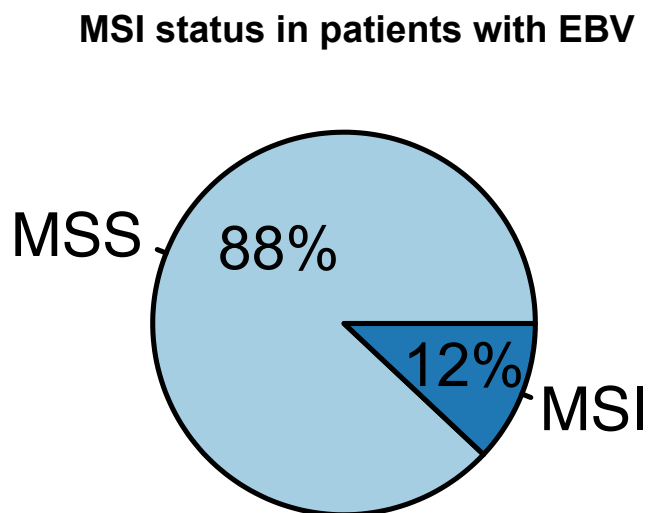

Supplement: Supplemental Information 1 — (A) Delta area of the consensus clustering. (B) Consensus cumulative distribution function (CDF) of the consensus clustering. (C) Progression-free survival curves of CIMP-H and CIMP-L subgroups. (D) MSI status in patients with EBV in CIMP-H subgroup. [file peerj-08-9624-s001.pdf]

A

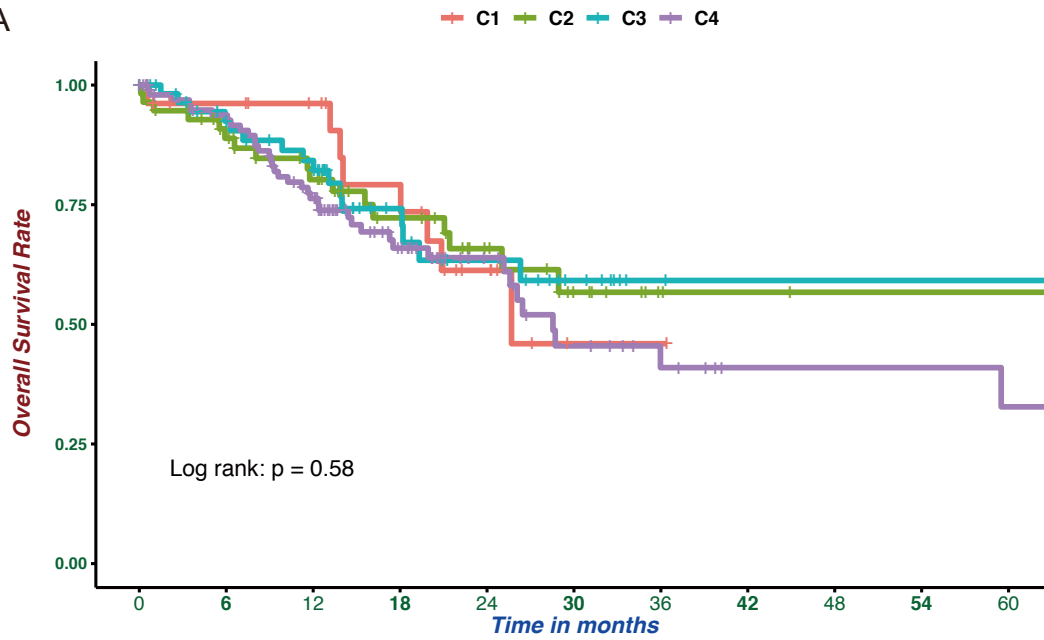

Number at risk

|   |     |    |    |    |    |    |   |   |   |   |   |
|---|-----|----|----|----|----|----|---|---|---|---|---|
| — | 27  | 22 | 19 | 14 | 7  | 1  | 1 | 0 | 0 | 0 | 0 |
| — | 57  | 45 | 36 | 25 | 16 | 9  | 3 | 2 | 1 | 1 | 1 |
| — | 58  | 47 | 40 | 21 | 15 | 9  | 3 | 2 | 2 | 2 | 2 |
| — | 102 | 88 | 66 | 38 | 23 | 14 | 9 | 5 | 5 | 5 | 4 |

B

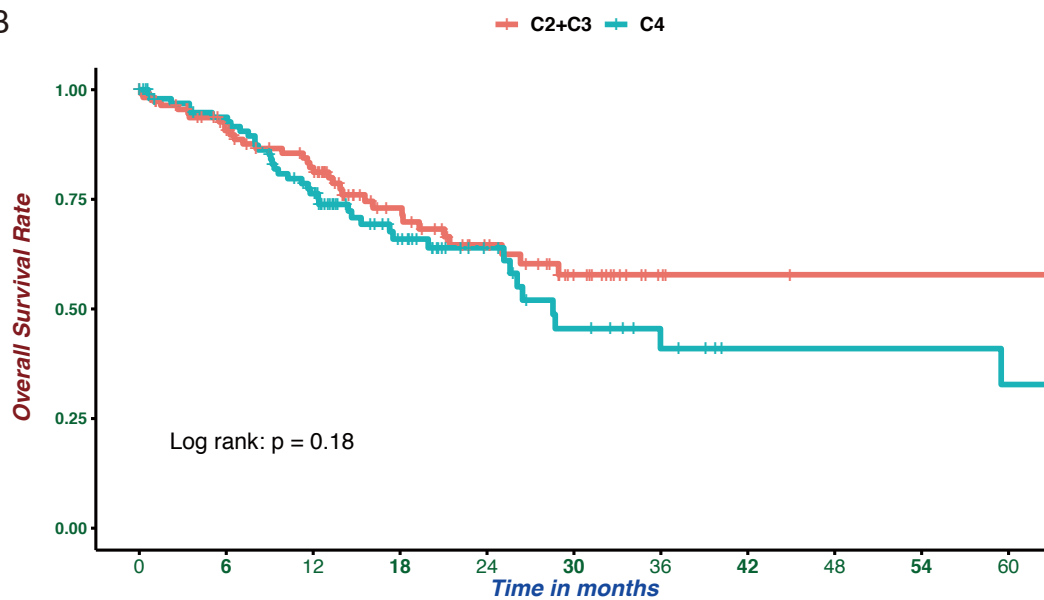

Number at risk

|   |     |    |    |    |    |    |   |   |   |   |   |
|---|-----|----|----|----|----|----|---|---|---|---|---|
| — | 115 | 92 | 76 | 46 | 31 | 18 | 6 | 4 | 3 | 3 | 3 |
| — | 102 | 88 | 66 | 38 | 23 | 14 | 9 | 5 | 5 | 5 | 4 |

Supplement: Supplemental Information 2 — (A) Kaplan-Meier survival curves of the C1, C2, C3 and C4 groups. The C1 group has the most prevalence of DNA hypermethylation and the C4 group has the lowest methylation level. The C2 and the C3 group have the medium methylation level. (B) Kaplan-Meier survival curves of the C2 + C3 and C4 groups. The C2 + C3 group represents the combination of the C2 and C3 groups. [file peerj-08-9624-s002.pdf]

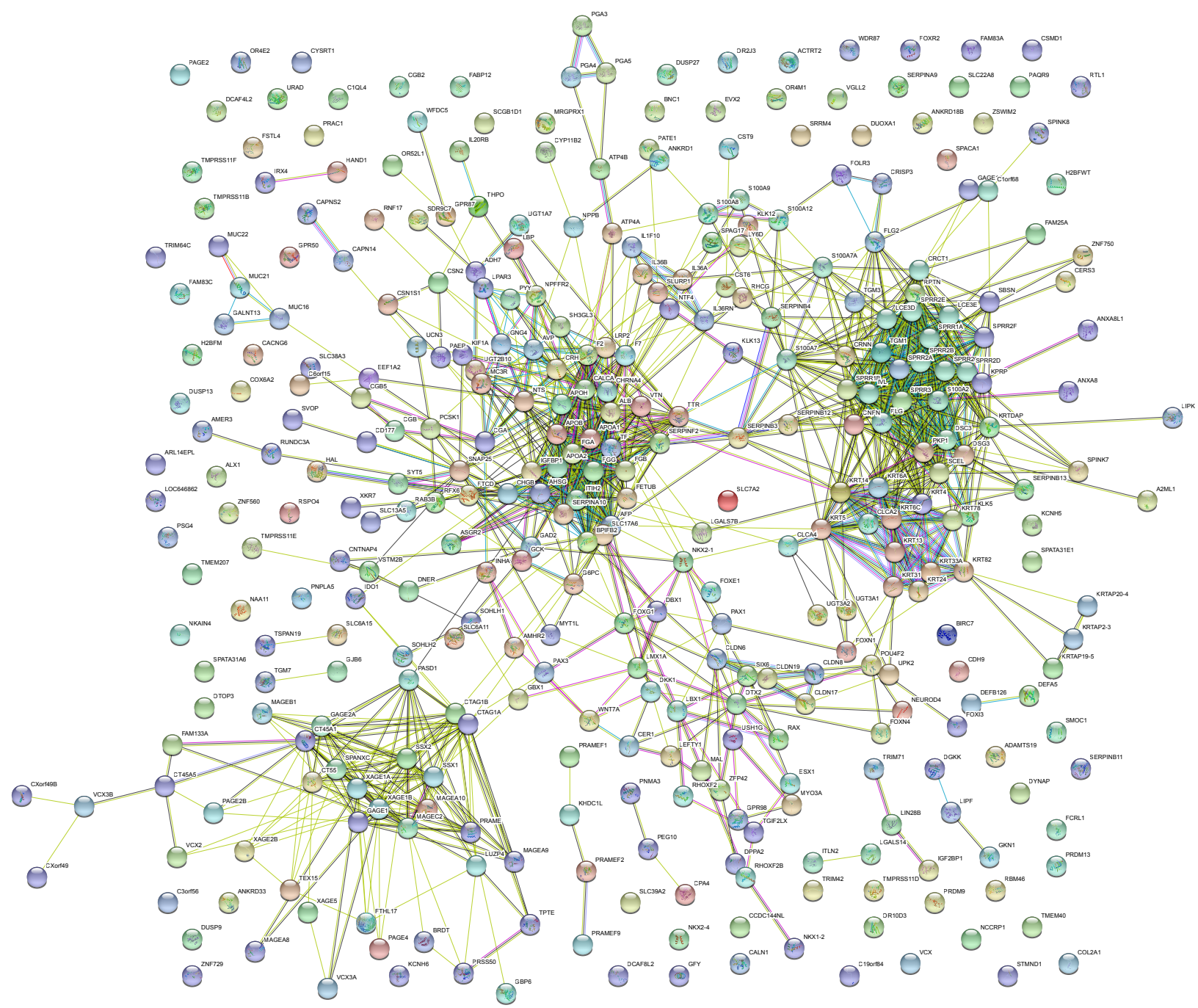

Supplement: Supplemental Information 3 — The PPI network was analyzed by String software. 366 nodes and 1176 edges are in the PPI network. [file peerj-08-9624-s003.pdf]
